# Phase retrieval via gain-based photonic XY-Hamiltonian optimization

**DOI:** 10.1038/s42005-026-02525-7

**Published:** 2026-02-03

**Authors:** Richard Zhipeng Wang, Guangyao Li, Silvia Gentilini, Davide Pierangeli, Marcello Calvanese Strinati, Claudio Conti, Natalia G. Berloff

**Affiliations:** 1 Department of Applied Mathematics and Theoretical Physics, University of Cambridge, Cambridge, UK; 2 Department of Physics, Sapienza University of Rome, Rome, Italy; 3 Research Center Enrico Fermi, Rome, Italy

**Keywords:** Applied optics, Applied mathematics, Information theory and computation

## Abstract

Phase-retrieval from coded diffraction patterns (CDP) is important to X-ray crystallography, diffraction tomography and astronomical imaging, yet remains a hard, non-convex inverse problem. We show that CDP recovery can be reformulated exactly as the minimization of a continuous-variable XY Hamiltonian and solved by gain-based photonic networks. The coupled-mode equations we exploit are the natural mean-field dynamics of exciton-polariton condensate lattices, coupled-laser arrays and driven photon Bose-Einstein condensates, while other hardware such as the spatial photonic Ising machine can implement the same update rule through high-speed digital feedback, preserving full optical parallelism. Numerical experiments on images, two- and three-dimensional vortices and unstructured complex data demonstrate that the gain-based solver consistently outperforms the state-of-the-art Relaxed-Reflect-Reflect (RRR) algorithm in the medium-noise regime (signal-to-noise ratios 10-40 dB) and retains this advantage as problem size scales. Because the physical platform performs the continuous optimisation, our approach promises fast, energy-efficient phase retrieval on readily available photonic hardware.

## Abstract (web summary)

Recovering missing phase from measured intensity is central to X-ray crystallography and diffraction imaging but is a hard, non-convex inverse problem. The authors reformulate coded diffraction pattern phase retrieval exactly as minimizing a continuous XY energy and solve it with gain-based photonic oscillator networks with efficient scaling and noise tolerance.

## Introduction

Recently, there has been a rising interest in using physics-inspired, physics-based computing systems for solving hard optimization problems, including many that are NP-hard^1–6^. An important example is the minimization of the *XY* Hamiltonian with sign-varying couplings between spins, where each spin is allowed to take on a continuous value in the vector ${{{\bf{s}}}}_{i}\,=(\cos {\theta }_{i},\sin {\theta }_{i})$ or complex form ${s}_{i}={{{\rm{e}}}}^{{{\rm{i}}}{\theta }_{i}},$ where *θ*_*i*_ ∈ [0,  2*π*). Such continuous-spin systems appear naturally in gain-dissipative photonic lattices, exciton–polariton networks, laser arrays, and related platforms, where each oscillator is represented by a complex variable whose amplitude and phase evolve in time^7–12^.

The *XY* minimization problem involves the quadratic Hamiltonian

1 $${H}_{{XY}}(\{{s}_{i}\})\,=\,-\frac{1}{2}\displaystyle \mathop{\sum }\limits_{i,j}^{N}{J}_{{ij}}\,{s}_{i}\,{s}_{j}^{\ast }+\,{{\rm{c}}}.{{\rm{c}}}.,$$

where *J*_*ij*_ represents the pairwise couplings. The task is to find the spin configuration {*s*_*i*_} that minimizes *H*_*XY*_. This is a continuous quadratic optimization (QCO) problem, with applications ranging from clustering^10^ to portfolio optimization^13^.

One key motivation for studying *XY*-type physical networks is their ability to perform highly parallel, analog searches for low-energy configurations, thereby offering an alternative to purely digital algorithms. Recently, there have also been efforts to force an *XY*-based system into effectively binary (Ising-like) states by introducing a large penalty term. Specifically, one adds

2 $${H}_{{{\rm{P}}}}(\{{s}_{i}\})\,=\,{H}_{{XY}}\,+\,P\displaystyle \mathop{\sum }\limits_{i=1}^{N}[{s}_{i}^{\ast 2}+{{\rm{c}}}.{{\rm{c}}}.],$$

where *P* > 0 is chosen so as to penalize any non-zero real part of *s*_*i*_. If *P* is sufficiently large, spins tend to align at phases$\pm \pi/2$, effectively reducing the continuous-spin *XY* problem to the binary Ising problem.

The continuous nature of the *XY* Hamiltonian also makes it relevant to other QCO tasks. One such problem of practical importance is the phase-retrieval problem. This is typically stated as follows: given a real measurement vector ${{\bf{b}}}\in {{\mathbb{R}}}^{M}$ and a complex matrix ${{\bf{A}}}\in {{\mathbb{C}}}^{M\times N}$, one seeks to find a complex vector ${{\bf{x}}}\in {{\mathbb{C}}}^{N}$ satisfying

3 $$| {{\bf{A}}}\,{{\bf{x}}}| \,=\,{{\bf{b}}},$$

where ∣ ⋅ ∣ denotes element-wise amplitude. Its practical significance arises because this problem is often encountered in applications such as X-ray crystallography^14,15^, astronomical imaging^16^, and diffraction imaging^17,18^.

In many of these applications, the complex vector **x** represents the complete information about the sample and is referred to as the *sample vector*. The matrix **A** describes the action of the optical system, often well-approximated by a Fourier transform. While the intensity of the resulting electromagnetic wave can be measured by standard detectors (e.g. charge-coupled devices) to yield the real-valued amplitude **b**, the phase component is typically lost in the measurement process. Recovering **x** from **b** and **A** constitutes the *phase-retrieval problem*, which is NP-complete^19^, underscoring its computational difficulty.

Moreover, without further constraints, the phase-retrieval problem is frequently *ill-posed* because multiple distinct sample vectors **x** can give rise to the same measured amplitude **b**. For instance, if **A** is a square matrix representing a discrete Fourier transform, any arbitrary phase profile can be applied to **b** before performing an inverse Fourier transform, producing infinitely many valid solutions **x**. In such scenarios, even a theoretically exact algorithm may yield a recovered vector $\widetilde{{{\bf{x}}}}$ that deviates from the original **x**^20^. This means that any solution of the form **x** = **D**^(*i*)^**A**^−1^**b**, where **D**^(*i*)^ is a diagonal matrix with arbitrary diagonal elements of the form ${{{\rm{e}}}}^{{{\rm{i}}}{\theta }_{i}}$, *θ*_*i*_ ∈ [0, 2*π*) and **A**^−1^ is the inverse discrete Fourier transform matrix, will satisfy the requirement given by Eq. (3). Efforts to mitigate ill-posedness often rely on additional assumptions about the sample vector **x**. For instance, some approaches impose a sparsity constraint by specifying the number of nonzero entries in **x**^21^. Others adopt even stricter conditions, stipulating the exact support of **x**; that is, which elements are nonzero^22–25^. However, none of these methods can guarantee that the resulting phase-retrieval problem is well-posed (i.e. admits a unique solution).

Amongst the works that investigated phase-retrieval problems with support constraint, Tradonsky et al.^25^ proposed a non-digital solver, namely a cavity laser, to solve small-scale phase-retrieval problems. Their use of an analog physical device to solve phase-retrieval problems is similar to the spirit of this paper. However, the proposed laser solver required an aperture that reproduced the known support of the 2D sample object, which limited the scalability and applicability of the solver to larger-sized and non-trivially shaped sample objects or 3D sample objects.

**Fig. 1:**
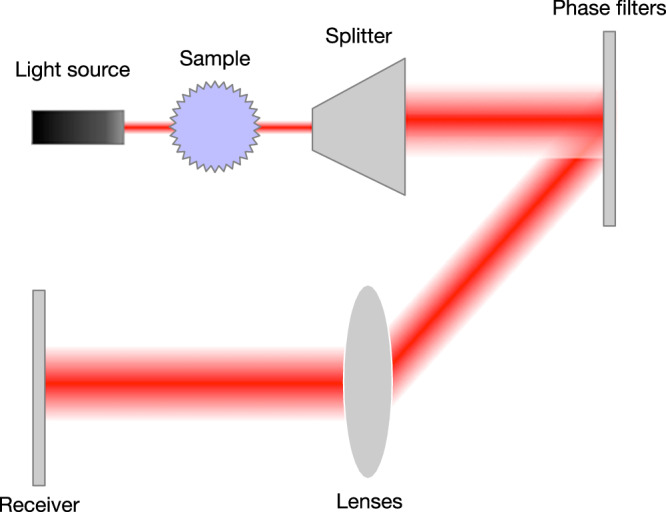
A schematic diagram for the CDP experiment framework. A light source is diffracted by the sample under investigation, and the diffracted complex-valued signal is split into *L* identical beams, each of which is directed towards a phase filter that modifies the phase of the incident signal at each spatial location. The phase-modified beams are then directed through a lens system, and their intensities are finally captured.

Candes et al.^26^ proposed a formulation called the “coded diffraction pattern” (CDP) experiment, which provides some solution uniqueness guarantee. Under this formulation, a sample vector **x** is first subjected to the action of *L* random phase filters, each defined by a diagonal matrix **D**^(*i*)^, *i* = 1, …, *L*. Each filter is defined by diagonal entries that are complex numbers with unit magnitude and randomly distributed phases. Thus, the filter modifies only the phase of the incoming signal while leaving its amplitude unchanged. This produces *L* filtered sample vectors, which are Fourier transformed separately. The results are then concatenated, and amplitudes are taken to form the observation vector **b**. Hence, in this formulation, **A** is an *M* by *N* complex matrix, where *M* = *L* × *N*, and represents the combined action of the random filters and the Fourier transforms. Figure 1 provides a schematic diagram showing the experimental setup of this arrangement. This formulation is theoretically appealing because if the distribution of the random diagonal matrix elements satisfies certain conditions, then with sufficiently large *L*, the solution of the phase-retrieval problem is guaranteed to be unique^26^. It was also demonstrated numerically that even with filters whose diagonal matrix elements did not follow random distributions that satisfy the conditions laid out in ref. ^26^, the problem could still become well-posed with increased *L*. Empirically, the process of applying multiple filters corresponds to the practice of oversampling the target and is reasonably achievable in many imaging applications^26–28^.

The performance of conventional phase-retrieval algorithms in this setting has been systematically examined in ref. ^27^. More recent proposals include semidefinite programming^29,30^, matrix completion^31^, and an alternating-projection method called *Relaxed-Reflect-Reflect* (RRR)^32^. Although RRR lacks a comprehensive theoretical foundation, subsequent investigations^33^ reported robust empirical performance. In particular, ref. ^34^ demonstrated that RRR outperforms both the “Phase Lift” approach of Candès et al.^29^ and Wirtinger flow methods^35^ in a variety of benchmark tests. Another approach, making use of unsupervised learning called “DeepPhaseCut” was also proposed^36^, and was shown to also outperform “Phase Lift” and “Phase Cut” in terms of computational speed. This approach demonstrated the possibility of combining machine learning techniques with traditional phase-retrieval algorithms for better computational efficiency.

In this work, we demonstrate that the CDP variant of phase retrieval can be reformulated as an *XY* Hamiltonian minimization problem, amenable to physical solvers that emulate the same Hamiltonian, for instance, through gain-based oscillator networks^3,37^. Under moderate noise levels in the measured amplitudes, we show numerically that a gain-based system can outperform the state-of-the-art Relaxed-Reflect-Reflect (RRR) algorithm and reliably reconstruct complex-valued experimental data with high accuracy.

## Methods

### Mapping phase retrieval into *XY* problem

The work of Waldspurger et al.^30^ first recast phase retrieval as a non-convex quadratic program, facilitating its solution via a suitable relaxation method. Following a similar line of reasoning, we show how to reformulate phase retrieval in the CDP experiment setting as an *XY* Hamiltonian minimization problem.

We start by defining the unknown phase of the observation as **p**, where all elements of this vector are complex and have unit amplitude, i.e., ${p}_{i}\in \{{{{\rm{e}}}}^{{{\rm{i}}}{\theta }_{i}}|{\theta }_{i}\in [0,2\pi )\}$. By this definition, we have

4 $$\mathop{\sum }\limits_{j=1}^{N}{A}_{ij}{x}_{j}={b}_{i}{p}_{i}\,\,{{\rm{for\; all}}}\,i=1,\ldots ,M{{\rm{}}}.$$

Suppose that the complete observation information including the phase information **p** and the amplitude information **b** is known, then the process of trying to find **x** that satisfy the constraint given by Eq. (4) is equivalent to the minimization of the cost function ${{\mathscr{E}}}$ given by

5 $${{\mathscr{E}}}(\{{x}_{j}\})=\mathop{\sum }\limits_{i=1}^{M}{\left|\mathop{\sum }\limits_{j=1}^{N}{A}_{ij}{x}_{j}-{b}_{i}{p}_{i}\right|}^{2},$$

where the minimization is over all possible sets of {*x*_*j*_} for *j* ∈ {1, ⋯  , *N*}. If the observations **b** and **p** are exact, the minimum value of this expression should be 0, but if uncertainty exists in either of them, then this expression is a least-squares problem over **x**. The solution to the least-square problem is given by the Moore–Penrose inverse, also known as the pseudoinverse of **A**, denoted as **A**^†^:

6 $${x}_{i}=\mathop{\sum }\limits_{j}^{M}{A}_{ij}^{{\dagger} }{b}_{j}{p}_{j}$$

Hence, for any given set of observation phase {*p*_*i*_}, the minimal cost function ${{\mathscr{E}}}$ is given by

7 $${{\mathscr{E}}}(\{{p}_{i}\})=\mathop{\sum }\limits_{i}^{M}{\left|\mathop{\sum }\limits_{j}^{N}\mathop{\sum }\limits_{k}^{M}{A}_{ij}{A}_{jk}^{{\dagger} }{b}_{k}{p}_{k}-{b}_{i}{p}_{i}\right|}^{2}$$

This means that to solve the original phase-retrieval problem, one needs to find a set of {*p*_*i*_} that minimizes this cost function ${\mathscr{E}}$, which is essentially a QCO problem over a set of variables {*p*_*i*_} that all have unit amplitude and unconstrained phases, so this problem can be mapped into an *XY* Hamiltonian minimization problem.

To put it into an explicit *XY* Hamiltonian form, we rearrange Eq. (7) as follows:

8 $${{\mathscr{E}}}(\{{p}_{i}\}) 	 = \mathop{\sum }\limits_{i}^{M}{\left|\mathop{\sum }\limits_{j,k}^{N,M}{A}_{ij}{A}_{jk}^{{\dagger} }{b}_{k}{p}_{k}-{\sum }_{k}^{M}{\delta }_{ik}{b}_{k}{p}_{k}\right|}^{2}\\ 	 = \mathop{\sum }\limits_{i}^{M}{\left|\mathop{\sum }\limits_{k}^{M}\left(\mathop{\sum }\limits_{j}^{N}{A}_{ij}{A}_{jk}^{{\dagger} }-{\delta }_{ik}\right){b}_{k}{p}_{k}\right|}^{2}\\ 	 = \mathop{\sum }\limits_{i}^{M}{\left|\mathop{\sum }\limits_{k}^{M}{G}_{ik}{b}_{k}{p}_{k}\right|}^{2}\\ 	 = \mathop{\sum }\limits_{i}^{M}\mathop{\sum }\limits_{jk}^{M}{G}_{ij}{G}_{ki}^{* }{b}_{j}{p}_{j}{b}_{k}{p}_{k}^{* }\\ 	 = -\mathop{\sum }\limits_{jk}^{M}{\widetilde{J}}_{jk}{p}_{j}{p}_{k}^{* },$$

where from the second line to the third line for ease of notation we defined ${G}_{ik}={\sum }_{j}^{N}{A}_{ij}{A}_{jk}^{{\dagger} }-{\delta }_{ik}$, and from the fourth line to the last we identified the coupling matrix elements of the equivalent *XY* Hamiltonian to be ${\widetilde{J}}_{jk}=-{\sum }_{i}^{M}{G}_{ij}{G}_{ki}^{* }{b}_{j}{b}_{k}$.

In principle, Eq. (8) is already in the form of an *XY* Hamiltonian we first introduced in Eq. (1). Noting that **AA**^†^ is Hermitian and **AA**^†^**AA**^†^ = **AA**^†^ by property of the pseudoinverse, one can simplify the expression for ${\widetilde{J}}_{ij}$ to

9 $${\widetilde{J}}_{ij}=\left(\mathop{\sum }\limits_{k}^{N}{A}_{ik}{A}_{kj}^{{\dagger} }-{\delta }_{ij}\right){b}_{i}{b}_{j},$$

which leads to an equivalent *XY* Hamiltonian:

10 $${{\mathscr{E}}}(\{{p}_{i}\}) 	 =-\mathop{\sum }\limits_{ij}^{M}\left({b}_{i}{b}_{j}\mathop{\sum }\limits_{k}^{N}{A}_{ik}{A}_{kj}^{{\dagger} }\right){p}_{i}{p}_{j}^{* }+\mathop{\sum }\limits_{ij}^{M}{\delta }_{ij}{p}_{i}{p}_{j}^{* }\\ 	={H}_{XY}(\{{p}_{i}\})+M,$$

where *M* is as before the dimension of observation vector **b**. Hence, minimizing ${{\mathscr{E}}}$ is equivalent to minimizing the simpler *XY* Hamiltonian *H*, whose coupling matrix elements are given by

11 $${J}_{ij}=\mathop{\sum }\limits_{k}^{N}{A}_{ik}{A}_{kj}^{{\dagger} }{b}_{i}{b}_{j}.$$

This coupling matrix was then used as input to the simulated gain-based system, with outcomes presented in the “Results” section.

We note that the Moore–Penrose pseudoinverse *A*^†^ may become ill-conditioned when rank(*A*) < *N*. However, as we will illustrate in the later subsection “Generation of CDP phase-retrieval problems”, the way that matrix **A** is built in the CDP phase-retrieval problem via Eq. (15) means that **A** is always well-conditioned and its pseudoinverse is exceptionally well-behaved. For completeness, we also tested a Tikhonov-regularized alternative,

$${(c{{\bf{I}}}+A{A}^{* })}^{-1}{A}^{* },$$

with *c* > 0, which can be used to stabilize the inversion. In practice, choosing $c \sim 1{0}^{-3}\parallel A{\parallel }_{2}^{\,2}$ leaves all numerical results within the error bars reported below.

### Simulated gain-based system

To minimize the *XY* Hamiltonian *H*_*XY*_ for a given phase-retrieval problem, we simulate an oscillator network following the gain-based dynamics given by

12 $$\frac{{{\rm{d}}}{\psi }_{i}}{{{\rm{d}}}t}=({\gamma }_{i}-|{\psi }_{i}{|}^{2}){\psi }_{i}+\displaystyle \mathop{\sum }\limits_{j}^{M}{J}_{{ij}}{\psi }_{j}$$

13 $$\frac{{{\rm{d}}}{\gamma }_{i}}{{{\rm{d}}}t}=\epsilon (1-|{\psi }_{i}{|}^{2}),$$

where ${\gamma }_{i}\in {\mathbb{R}}$ is the effective injection rate of oscillator *i* (gain minus losses), ${\psi }_{i}\in {\mathbb{C}}$ characterize each oscillator, and *J*_*ij*_ specifies the coupling strength between oscillators. *J*_*ij*_ is calculated from the given phase-retrieval problem according to our previous discussion. *ϵ* is an externally controlled positive constant, which measures the responsiveness of the gain of each oscillator to the amplitude variations of each oscillator. Eqs. (12) and (13) faithfully reproduce the gain-dissipative evolution observed in networks of exciton–polaritons hosted in semiconductor micro-cavities^38^, in coupled-laser arrays^12^, in driven photonic oscillator lattices^39^, and spin wave Ising machines^40^. Hardware platforms that do not possess intrinsic gain control, most notably the spatial photonic Ising machine (SPIM)^41,42^, can nevertheless implement the same update rule by applying digital feedback to the spatial-light modulator after each optical pass, thereby emulating the gain–loss loop in silico while retaining full optical parallelism. This kind of dynamics was formulated and studied in refs. ^3,8,43^, showing that the dynamics of this system lead to stationary states close to or at the global minimum of the XY Hamiltonian specified by the coupling matrix **J** with high probability. Equation (12) encapsulates the main dynamics of *ψ*_*i*_ with the interplay of the effective gain, non-linear loss, and the coupling terms. Eq. (13) provides a feedback mechanism that pushes the amplitudes of all oscillators towards 1. It was previously reported that this feedback mechanism is crucial for the gain-dissipative dynamics to produce good solutions close to the true global minimum of the *XY* Hamiltonian^8^.

In principle, the gain-based optimizer works as follows. At the start of the dynamical evolution, the oscillator network has a set of highly negative effective gains *γ*_*i*_, so the system has a stable fixed point at *ψ*_*i*_ = 0. Due to the gain dynamics given by Eq. (13), the gains *γ*_*i*_ increase over time and eventually cross a critical value at which supercritical Hopf bifurcation occurs^3,43^. The *ψ*_*i*_ = 0 fixed point becomes unstable, and oscillators spontaneously increase to some non-zero amplitudes and start to have well-defined phases. Over time, all amplitudes ∣*ψ*_*i*_∣ approach 1, while the phases of oscillators also approach their stationary values, which will be read out as the solution to the *XY* problem.

In our simulations, we first initialized the amplitude of *ψ*_*i*_ to some random small but non-vanishing values uniformly distributed in the range (0, 0.1) and initialized their phase uniformly randomly in the range [0, 2*π*). Initial gains *γ*_*i*_ were initialized uniformly randomly in such a range of values, so that initial stages of the evolution are below the threshold, which means that oscillator amplitudes will tend to decay to 0 if gains are held constant at this level. In practice, this was achieved by selecting *γ*_*i*_ values uniformly randomly from the range [−*λ*_max_(**J**)−2,−*λ*_max_(**J**)−1) where *λ*_max_(**J**) denotes the maximum eigenvalue of coupling matrix **J**. Some preliminary simulations showed that the feedback coefficient *ϵ* mainly affected the rate of convergence to the final solution, but did not impact the quality of the final solution significantly, as long as the value of *ϵ* was not so large that the system would diverge. Hence, to show that the proposed gain-based dynamical system can solve a wide range of phase-retrieval problems without considerable hyper-parameter tuning beforehand on a per-problem basis, we simply used the value of *ϵ* = 1 throughout all simulations carried out in this paper. The system was then evolved with an adaptive timestep Runge–Kutta 4th-order method using the “Scipy” Python package until it reached a stationary state or a maximum simulation time was reached, and the phases of oscillators were used as the spin configurations **p** for the *XY* problem. This can then be substituted into Eq. (6) to find the solution **x** to the original phase-retrieval problem.

### Generation of CDP phase-retrieval problems

For a given sample vector ${{\bf{x}}}\in {{\mathbb{C}}}^{N}$, we had to generate an observation vector ${{\bf{b}}}\in {{\mathbb{R}}}^{M}$ under the CDP experiment framework to serve as the input to our *XY* minimizer. A set of *L* random filters was first generated. Each filter could be represented as a diagonal matrix **D**^(*i*)^ where *i* = 1, …, *L*, and each diagonal element ${D}_{jj}^{(i)}$ was uniformly randomly selected from {1, *i*, −1, −*i*}, corresponding to a phase shift of 0, *π*/2, *π*, 3*π*/2, respectively.

We represent the action of the Fourier transform on the sample vector by using the discrete Fourier transform (DFT) matrix defined as:

$${{{\bf{F}}}}_{N}=\frac{1}{\sqrt{N}}\left(\begin{array}{ccccc}1 & 1 & 1 & \cdots & 1\\ 1 & {\omega }_{N} & {\omega }_{N}^{2} & \cdots & {\omega }_{N}^{N-1}\\ 1 & {\omega }_{N}^{2} & {\omega }_{N}^{4} & \cdots & {\omega }_{N}^{2(N-1)}\\ \vdots & \vdots & \vdots & \ddots & \vdots \\ 1 & {\omega }_{N}^{N-1} & {\omega }_{N}^{2(N-1)} & \cdots & {\omega }_{N}^{{(N-1)}^{2}}\end{array}\right),$$

where *ω*_*N*_ = e^−2*πi*/*N*^ is the *N*th root of unity.

One set of observation from one phase filter can be obtained by multiplying **F**_*N*_ and **D**^(*i*)^ with **x**, so part of the complex-valued matrix **A** can be defined as:

14 $${{{\bf{A}}}}^{(i)}={{{\bf{F}}}}_{N}{{{\bf{D}}}}^{(i)}$$

Matrix ${{{\bf{A}}}}^{(i)}\in {{\mathbb{C}}}^{N\times N}$ can be stacked columnwise to obtain the complete matrix **A** as illustrated below:

15 $${{\bf{A}}}=\left(\begin{array}{c}{{{\bf{A}}}}^{(1)}\\ {{{\bf{A}}}}^{(2)}\\ \vdots \\ {{{\bf{A}}}}^{(L)}\end{array}\right).$$

This matrix ${{\bf{A}}}\in {{\mathbb{C}}}^{M\times N}$, together with **x**, can be substituted into Eq. (3) to produce the observation vector **b**. The observation vector **b** and the generated matrix **A** define this CDP phase-retrieval problem, and they are supplied to our gain-based optimizer Eqs. (12) and (13), and the phases *θ*_*i*_ are found as the stationary states. The sample vector **x** is then reconstructed from Eq. (6).

We note that because each block **A**^(*i*)^ is unitary, the conjugate transpose of **A**, denoted as A^*^, has the following property:

16 $${{{\bf{A}}}}^{* }{{\bf{A}}}=\mathop{\sum }\limits_{i=1}^{L}{\left({{{\bf{A}}}}^{(i)}\right)}^{* }{{{\bf{A}}}}^{(i)}=L{{\mathbb{I}}}_{N}$$

where ${{\mathbb{I}}}_{N}$ is the identity matrix of dimension *N*. Thus, the pseudoinverse of **A** simplifies to ${{{\bf{A}}}}^{{\dagger} }=\frac{1}{L}{{{\bf{A}}}}^{* }$. Hence, **A** has full column rank and condition number 1, so its pseudoinverse is always well-behaved.

The block stacking of all Fourier operators **F** and diagonal masks **D**^(*i*)^ is mathematically identical to the decomposition of a fully connected interaction matrix into multiple Mattis subproblems recently realized in a fully programmable SPIM via focal-plane division, where the energies of all sub-Hamiltonians are computed in parallel on distinct camera regions^42^. This analogy suggests that an experimental CDP phase-retrieval setup could exploit the same optical parallelism, processing the *L* masks in a single physical shot and obtaining an *L*-fold reduction in acquisition time.

Additionally, by substituting ${{{\bf{A}}}}^{{\dagger} }=\frac{1}{L}{{{\bf{A}}}}^{* }$ into Eq. (11), the coupling matrix **J** that needs to be implemented in hardware can be expressed as

17 $${{\bf{J}}}=\frac{1}{L}\,{{\rm{diag}}}\,({{\bf{b}}}){{\bf{A}}}{{{\bf{A}}}}^{* }\,{{\rm{diag}}}\,({{\bf{b}}})$$

where diag(**b**) denotes a diagonal matrix whose elements are elements of vector **b**. This implies that although matrix **J** has dimension *M* × *M* and is dense, its rank is at most *N*. This low-rank structure can be exploited in photonic hardware such as SPIM, which natively implements rank-one or low-rank interaction matrices via optical propagation^13^.

In this study, we also considered the case where **b** is noisy, which is to be expected in realistic experimental data. In this case, a noisy observation vector $\widetilde{{{\bf{b}}}}$ is produced by adding a normally distributed random noise to each element of the noiseless observation vector **b**, i.e. ${\widetilde{b}}_{i}={b}_{i}+{\xi }_{i}$ with ${\xi }_{i} \sim {{\mathcal{N}}}(0,{\sigma }^{2})$. The variance of noise *σ* was used to control the magnitude of noise in the observational data so that we could investigate its impact on the performance of the gain-based system in solving the phase-retrieval problem. To quantify the amount of noise in the given noisy observation vector, we define the signal-to-noise ratio (SNR) as follows:

18 $$\,{{\rm{SNR}}}\,=10{\log }_{10}\frac{\parallel {{\bf{b}}}{\parallel }_{2}}{\parallel \widetilde{{{\bf{b}}}}-{{\bf{b}}}{\parallel }_{2}},$$

which is conventionally measured on a logarithmic scale and quoted in units of decibels. In this expression, ∥ ⋅ ∥_2_ denotes the vector 2-norm.

Additionally, we note that beyond Gaussian noise, Poisson noise and systematic errors are also often present in realistic diffraction experiments. To investigate the robustness of our proposed phase-retrieval method against Poisson noise, we also generate noisy observation vector $\widetilde{{{\bf{b}}}}$ whose elements are given by ${\widetilde{b}}_{i}={B}_{i}/n$, where random variable *B*_*i*_ ~ *P*(*λ* = *nb*_*i*_) (i.e. it follows Poisson distribution with *λ* given by *nb*_*i*_). The coefficient *n* controls the strength of the Poisson noise and thus allows us to generate a noisy signal with the required SNR. To understand the impact of systematic error, we generate a noisy observation vector $\widetilde{{{\bf{b}}}}$ with a constant shift from the true value, which means its elements ${\widetilde{b}}_{i}={b}_{i}+n$, where *n* > 0 is the same for all *i* and controls the SNR of the resultant observation vector.

### Performance evaluation

To measure the quality of the calculated solution, we used metrics that were also used previously in refs. ^30,34^. The most direct metric to measure the success of the phase-retrieval algorithm is the Euclidean distance between the observation vector calculated from the recovered sample vector $\widetilde{{{\bf{x}}}}$ and the given observation vector **b**, normalized over the vector norm of the observation vector **b**.

For clearer visual representation, it is more convenient to express this quantity on a logarithmic scale, similar to SNR. Therefore, we define the relative observation error (ROE) in decibels as

19 $$\,{{\rm{ROE}}}\,=10{\log }_{10}\frac{\parallel | {{\bf{A}}}\widetilde{{{\bf{x}}}}| -{{\bf{b}}}{\parallel }_{2}}{\parallel {{\bf{b}}}{\parallel }_{2}},$$

where ∣ ⋅ ∣ denotes taking the amplitude element-wise, and $\widetilde{{{\bf{x}}}}$ is the sample vector calculated by the phase-retrieval algorithm. In experiments, the true sample vector **x** is typically unavailable, so the ROE would be an appropriate way to evaluate the quality of the recovered solution. However, in generated datasets where the true sample vector **x** is known a priori, we can instead measure the Euclidean distance between the recovered sample vector $\widetilde{{{\bf{x}}}}$ and the true sample vector **x**. Note that the recovered sample vector $\widetilde{{{\bf{x}}}}$ may have a global phase shift relative to **x** while reproducing the exact same observation vector **b**. Hence, the error metric should be defined as the minimum Euclidean distance between ${{{\rm{e}}}}^{{{\rm{i}}}\theta }{{{\bf{x}}}}^{ \sim }$ and **x** for all *θ* ∈ [0, 2*π*). This quantity is also expressed in decibels and is referred to as the relative sample error (RSE), which is defined as

20 $$\,{\mathrm{RSE}}\,=10{\log }_{10}\left({min}_{\theta }\frac{\parallel {{{\rm{e}}}}^{{{\rm{i}}}\theta }{{{\bf{x}}}}^{ \sim }-{{\bf{x}}}{\parallel }_{2}}{\parallel {{\bf{x}}}{\parallel }_{2}}\right).$$

In a phase-retrieval problem, this error is usually the most important quantity because it measures how close the recovered solution is to the original sample.

**Fig. 2:**
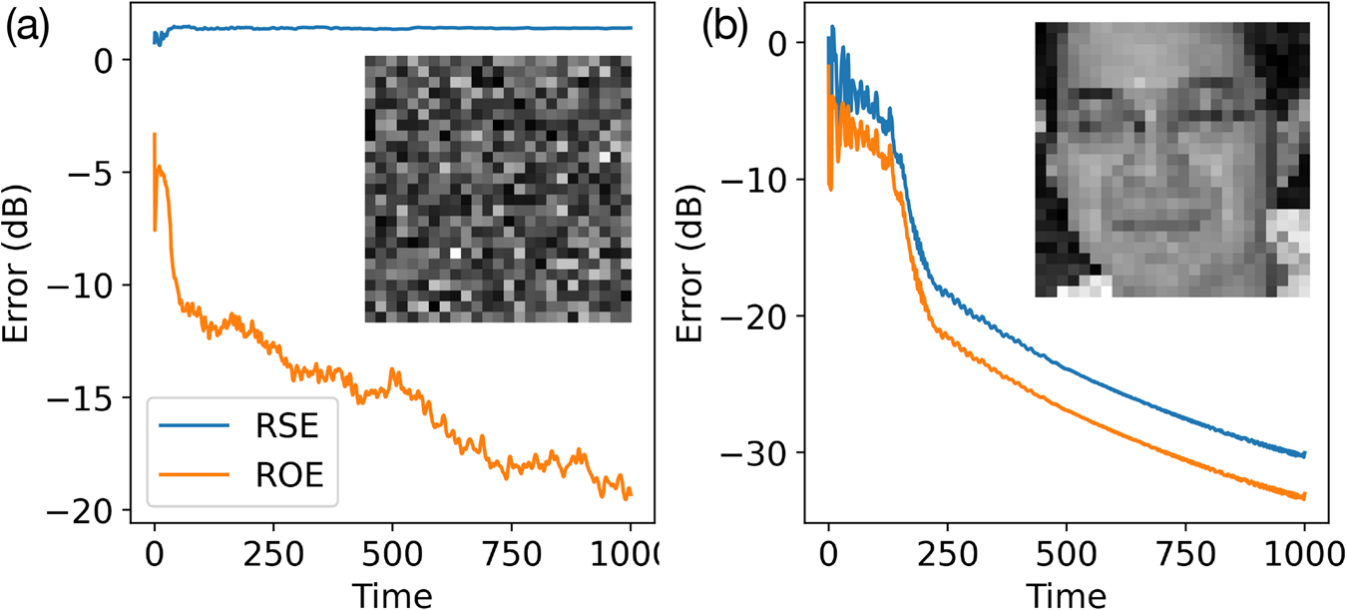
Comparison of phase-retrieval problems with different number of phase masks. **a** Time evolution of errors for the phase-retrieval problem under the CDP experiment framework with *L* = 2 phase masks. The inset gives the final reconstructed image produced by the phase-retrieval algorithm. **b** Time evolution of errors for the phase-retrieval problems with 5 phase masks. In both cases, the problems were solved with the gain-dissipative system with a random initial condition. The original image, which is visually identical to the image shown in the inset of (**b**), is from the labeled face in the wild (LFW) dataset^51^. See table “Fig. 2” in supplementary data.

The two error metrics are closely related but not equivalent, and both are required to give a complete picture of the well-posedness of the phase-retrieval problem itself and the performance of the phase-retrieval algorithm used. For example, if a solution calculated by a phase-retrieval algorithm yields a small ROE value but a large RSE value, this indicates that the algorithm is performing well, but the problem itself is poorly defined because it has more than one degenerate ground state (i.e., more than one **x** can all produce the same observation vector **b**). This is because the phase-retrieval algorithm only has access to **b** and **A**, so any solution $\widetilde{{{\bf{x}}}}$ that can minimize $\parallel | {{\bf{A}}}\widetilde{{{\bf{x}}}}| -{{\bf{b}}}{\parallel }_{2}$ is equally good to the algorithm, even if it might be far from the true **x** from which the problem was first constructed. This situation is illustrated in Fig. 2a, where the simulated gain-based system tries to recover an image from an observation vector produced by 2-phase filters. While ROE keeps decreasing, RSE has remained largely flat, and the system failed to recover the original image. As far as the phase-retrieval method is concerned, it is performing well because it is able to find a vector $\widetilde{{{\bf{x}}}}$ that produces an observation vector $\widetilde{{{\bf{b}}}}$ that is very close to the known observation vector **b**. This means that this phase-retrieval problem under the CDP experiment framework with only 2 phase masks is ill-defined, because it has degenerate ground states.

When both RSE and ROE are small, it suggests that the phase-retrieval problem is well defined and the solution found is close to the true solution. This case is shown in Fig. 2b, where the same gain-dissipative system recovers the same image from an observation vector produced by 5 phase filters. RSE and ROE decrease in tandem, indicating that the algorithm is approaching the planted ground state. As far as we know, there is no explicit formula which can give a *L* value for a given class of sample vector **x** such that it is guaranteed that the generated phase-retrieval problem will always have a unique solution^26^. It is down to empirical trial-and-error to determine what is the minimum value of *L* such that the generated phase-retrieval problem has a unique solution. Hence, when studying phase-retrieval algorithms, it is crucial to consider both the ROE and RSE to determine whether the phase-retrieval method itself or the problem at hand is responsible for the failure to recover the original signal. In all of the numerical experiments carried out in this paper, we incremented *L* until the difference between ROE and RSE remained small throughout the entire simulation duration, and the reconstruction result became visually faithful. We then used the smallest such *L* in all subsequent tests, but we also always checked both ROE and RSE for each class of problems we investigated to ensure that all random problem instances we generated resulted in a well-posed CDP phase retrieval problem.

### Comparison with existing algorithms

To compare the gain-dissipative system with established phase-retrieval methods, we focus on the RRR algorithm for phase retrieval^32^. Originally designed for the sparse variant of phase retrieval, RRR belongs to the family of alternating projection methods, similar to classic techniques such as the Gerchberg–Saxton (GS) method^44^, Fienup’s hybrid input–output (HIO) scheme^22^, and the shrinkwrap algorithm^45^. These algorithms operate in an iterative fashion, applying two distinct projection operators in sequence at each iteration. Among them, RRR has shown particularly strong empirical performance, surpassing multiple modern phase-retrieval methods^34^.

The RRR algorithm starts with a guessed observation vector **b**_0_, and employs two projections *P*_1_ and *P*_2_. When given a (generally complex) estimated observation vector **b**_*n*_, *P*_1_ keeps only the *S* largest elements in the vector and sets other to 0, where *S* is the given sparsity constraint in the observation data; For *P*_2_, when given ${{{\bf{b}}}}_{n}\in {{\mathbb{C}}}^{M}$, it keeps the phase of each element but overwrites their amplitudes with the known correct amplitudes in ${{\bf{b}}}\in {{\mathbb{R}}}^{M}$. Overall, in each iteration, the algorithm applies *P*_1_ and *P*_2_ as follows:

21 $${{{\bf{b}}}}_{n+1}={{{\bf{b}}}}_{n}+\beta [{P}_{2}(2{P}_{1}({{{\bf{b}}}}_{n})-{{{\bf{b}}}}_{n})-{P}_{1}({{{\bf{b}}}}_{n})],$$

where *β* is a constant parameter. We found the value *β* = 0.5 used by authors of Elser et al. ^34^ generally produced good results.

To adapt RRR for the CDP variant of the phase-retrieval problem, we follow the method proposed in ref. ^34^ and modify projection *P*_1_ to the following:

22 $${P}_{1}({{{\bf{b}}}}_{n})={{\bf{A}}}{{{\bf{A}}}}^{{\dagger} }{{{\bf{b}}}}_{n}.$$

One can motivate this projection by considering that **A**^†^**b**_*n*_ makes use of all *L* sets of observations, unique to the CDP formulation, to produce an “average” estimated sample vector **x**_*n*_ based on all available observations, and then using this best estimation to produce the next **b**_*n*+1_ by calculating **Ax**_*n*_, which in turn ensures that **b**_*n*+1_ produced this way remains in the range of **A**. We then substituted this modified projection *P*_1_ into the iterative scheme of Eq. (21), keeping the original *P*_2_ operator and *β* = 0.5, and applied the resulting RRR method to the same phase-retrieval instances used by the gain-based system.

The classical GS method, which was one of the first proposed heuristic methods for solving phase-retrieval problems, used the same *P*_1_ and *P*_2_ projections, but a simpler iterative formula:

23 $${{{\bf{b}}}}_{n+1}={P}_{2}({P}_{1}({{{\bf{b}}}}_{n})).$$

This iterative heuristic can also be applied to CDP phase-retrieval problems with modified projection *P*_1_ given by Eq. (22). The quality of solutions found by these methods was measured by the performance metrics presented in the “Performance evaluation” section.

In summary, for each class of sample vectors **x** considered in the “Results” section, we first generated matrix **A** with Eqs. (14) and (15), and then generated observation vector **b** with Eq. (3). Matrix **A** and vector **b** completely define the CDP phase-retrieval problem. To solve this problem with the simulated gain-based method, matrix **A** and observation vector **b** were then substituted into Eq. (11) to obtain the coupling matrix **J**, and it was used to construct the dynamical system defined by Eqs. (12) and (13). After the dynamical system evolves until it reaches a stationary state or a maximum simulation duration, the final phases of the complex-valued state parameters {*ψ*_*i*_} can be read out. The phases can then finally be substituted into Eq. (6) to produce the reconstructed sample vector **x**. This solution was compared against the solution obtained by presenting matrix **A** and observation vector **b** to the RRR and GS methods.

To ensure fairness in each set of comparisons, initial conditions for the RRR method and the gain-based method were always generated from the same initial random guess of phases *θ*_*i*_, which were uniformly randomly chosen from the range [0, 2*π*). Angles {*θ*_*i*_} became the initial phases of the complex-valued state {*ψ*_*i*_} for the gain-based method, and $\{{b}_{i}{{{\rm{e}}}}^{{{\rm{i}}}{\theta }_{i}}\}$ became the initial guess for the RRR and GS methods. Simulation results are shown in the “Results” section.

## Results

### Performance with real-valued sample vectors

Many studies^18,24,34^ have evaluated phase-retrieval algorithms using images that are real and strictly positive, represented by two-dimensional matrices. Although such images offer a convenient test bed, they restrict **x** to real values only, whereas real-world diffraction data are generally complex. Nevertheless, these simple test cases already reveal key limitations of traditional phase-retrieval algorithms.

**Fig. 3:**
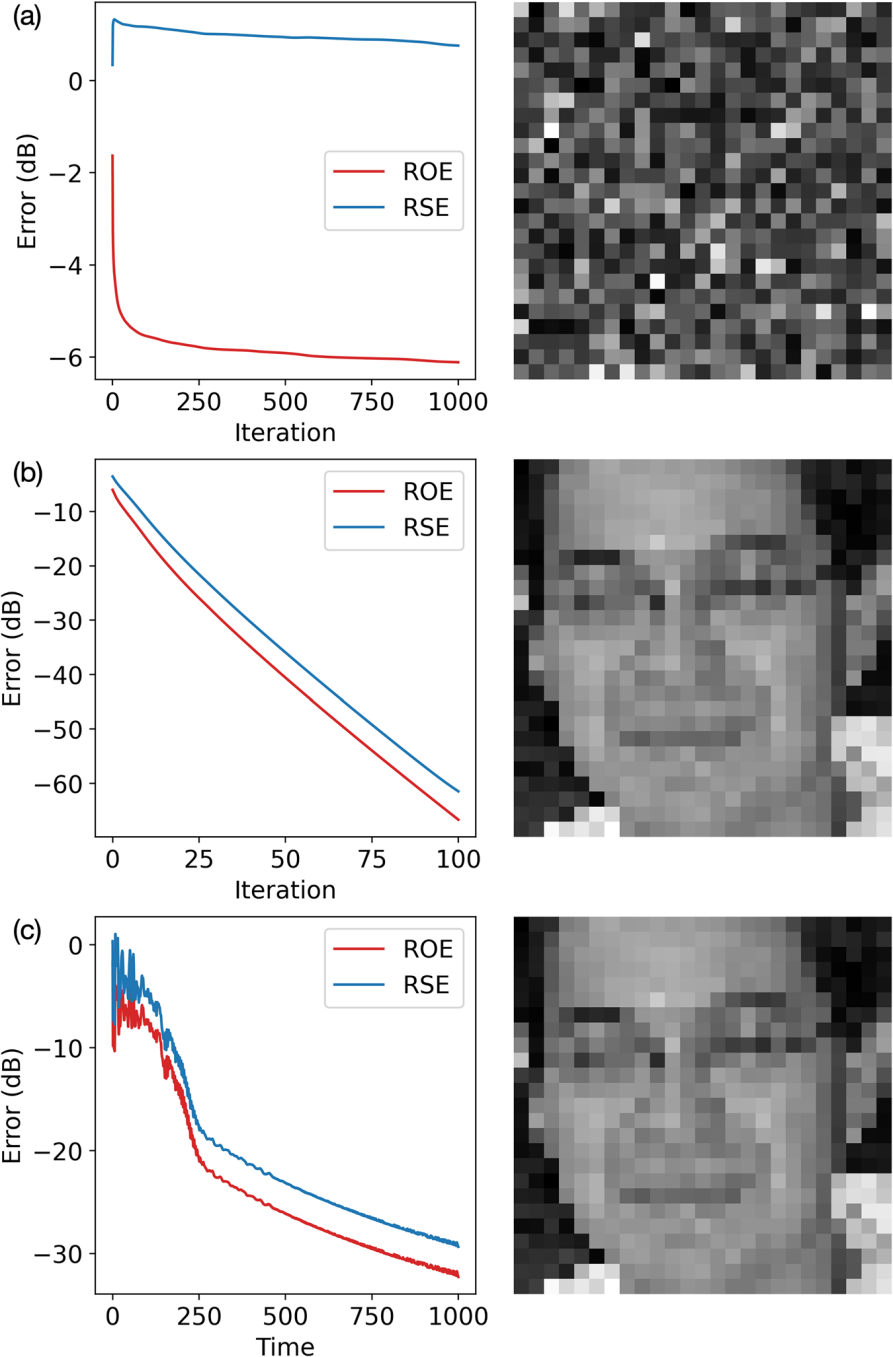
Comparison of the GS method and the gain-based system performance in recovering a real-valued image. **a** Error evolution and final reconstructed image with the GS method starting from a complex-valued random initial condition whose phase is uniformly randomly distributed in the range [0, 2*π*) and whose amplitude is the known observation vector **b**. **b** Error evolution and final reconstructed image with the GS method starting from an initial condition whose phase is obtained by multiplying **A** with a random real-valued vector $\widetilde{{{\bf{x}}}}$, and whose amplitude is the known observation vector **b**. **c** Error evolution and final reconstructed image with gain-based method starting from the exact same initial condition as used in (**a**). See “Fig. 3” table in supplementary data.

For instance, we used the positive real-valued image depicted in Fig. 2 to construct a CDP-based phase-retrieval problem with five phase filters. The resulting observation vector **b** was then presented to different solvers. As shown in Fig. 3a, when the GS method began from a random complex initialization for **b**, it quickly became stuck in a local minimum after about 1000 iterations, failing to reproduce any recognizable features of the original image. By contrast, starting from the same initial condition, the gain-dissipative system (Fig. 3c) followed a markedly different trajectory and produced significantly lower errors, ultimately reconstructing the image with high fidelity.

We further observed that a priori knowledge of **x** being real and positive can substantially simplify phase retrieval. For example, by multiplying **A** with a random positive real-valued vector $\widetilde{{{\bf{x}}}}$ and extracting its phase as the initial guess for GS, we obtained the error evolution and final reconstruction depicted in Fig. 3b. This carefully chosen initialization, which already exhibits a lower error than a random complex guess, allowed the GS method to recover the underlying image. Clearly, such information (i.e., that **x** is a non-negative real) makes the phase-retrieval problem more tractable. However, real-world experimental data generally produce complex-valued **x** without providing a straightforward initialization. Consequently, the GS method often encounters difficulty in practical scenarios. In the following section, we therefore focus on more general, complex-valued samples and benchmark the gain-based solver in that setting.

**Fig. 4:**
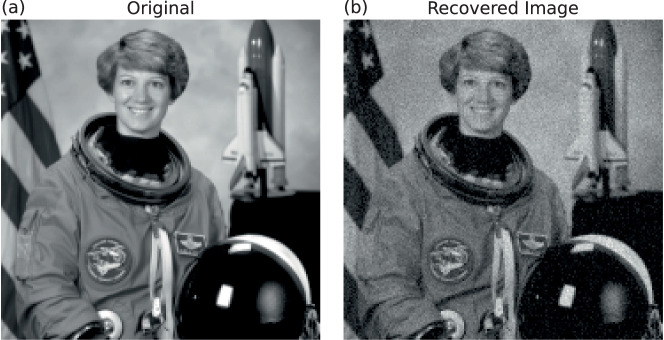
Phase retrieval with a large-scale sample vector using the gain-based system. **a** A 180 × 180 pixel grayscale image downsampled from the “astronaut” image of the “scikit-image” Python image dataset package^52^. This image is used as the sample vector in a CDP-based phase-retrieval setup with 8 phase filters, yielding an observation vector **b** of length 259,200. **b** The final reconstruction after the gain-based system evolves for *t* = 5 from a random initial condition. The resulting RSE is −9.4 and the ROE is −12.

As a final demonstration using real-valued data, Fig. 4a shows a high-resolution grayscale image of size 180 × 180, totaling 32,400 real-valued pixels. Despite this large problem dimension, the gain-based method successfully reconstructs the main features of the image after a short simulation (duration *t* = 5), as shown in Fig. 4b. Notably, this was achieved without leveraging the fact that the target sample vector is purely real. While the final RSE remains moderately high at −9.4, the essential image structure is clearly recognizable, albeit with visible background noise.

Although the gain-based method easily solves real-valued phase-retrieval problems, most practical applications involve recovering *complex*-valued data. Consequently, the remaining sections focus on benchmark cases with complex-valued samples to more accurately reflect real-world experimental conditions.

### Performance with complex-valued sample vectors

**Fig. 5:**
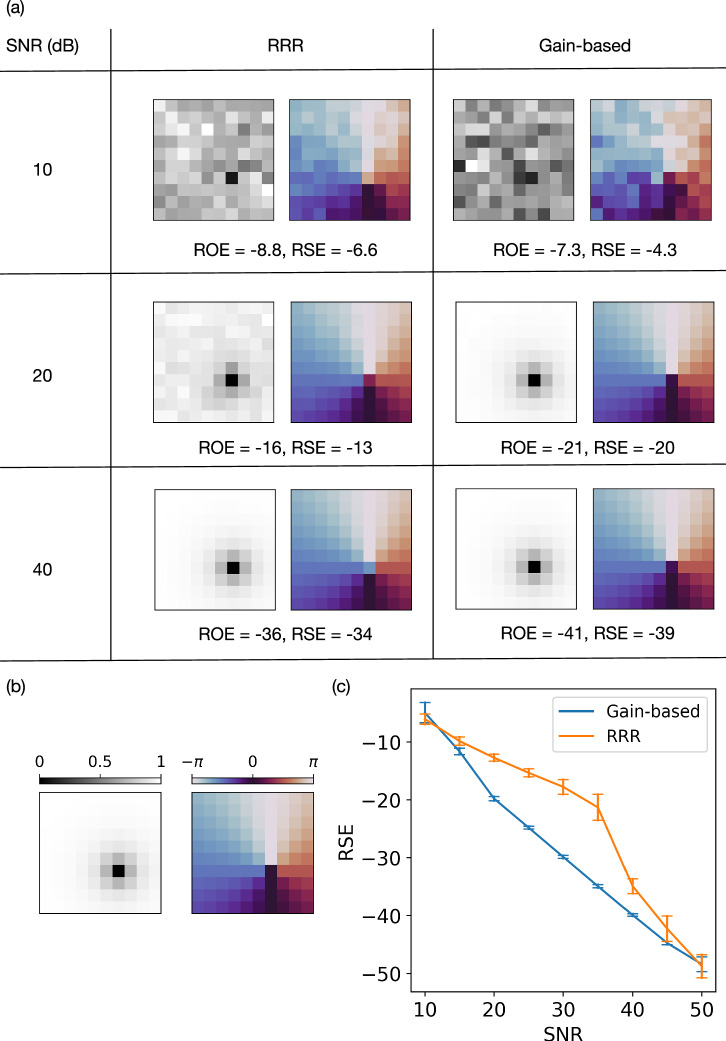
Phase retrieval of a two-dimensional vortex in the presence of noise, comparing RRR and the gain-based system. The phase-retrieval problems were constructed with *L* = 5 phase filters. The size of the vortex core *ξ* = 1, and the vortex field were discretised into a 10 × 10 grid. **a** Each panel displays the reconstructed sample vector $\widetilde{{{\bf{x}}}}$, where the grayscale image encodes amplitude, and the color image encodes phase. For each noise level, both RRR and the gain-based method start from the same initial condition. Here, RRR runs for 10,000 iterations, while the gain-based system is evolved to *t* = 1000. **b** The ground-truth sample vector **x** that describes a 2D vortex, showing amplitude (left) and phase (right). **c** Phase-retrieval error (RSE) versus the signal-to-noise ratio (SNR). In this test, the noisy observation vectors $\widetilde{{{\bf{b}}}}$ presented to the solvers contained Gaussian noise. Each data point represents the average of 20 random instances, where the vortex core is placed at different positions, and each algorithm is initialized randomly. Error bars denote the standard deviation of the final RSE values. See “Fig. 5” table in supplementary data.

We begin by investigating the reconstruction of a complex-valued image that can potentially be produced in an experiment: a two-dimensional vortex, where the vortex flow is given by the gradient of phase at each point in a plane. The field value at each point can be approximated by

24 $$v(x,y)=\frac{(x-{x}_{0})+i(y-{y}_{0})}{\sqrt{{(x-{x}_{0})}^{2}+{(y-{y}_{0})}^{2}+{\xi }^{2}}},$$

where (*x*_0_, *y*_0_) is the center of the vortex and *ξ* is the size of the vortex core^46^. The amplitude, ∣*v*∣ (greyscale), and phase of the vortex $\arg (v)$ (color) can be visualized as shown in Fig. 5b.

In most practical experiments, the observed amplitudes $\widetilde{{{\bf{b}}}}$ deviate from the ideal measurements **b** due to noise. As illustrated in Fig. 5a, both the RRR and gain-based solvers were presented with noisy observations injected with Gaussian noises of varying magnitudes. In the high-noise regime (small SNR values), neither method recovered a solution close to the original sample **x**, shown in Fig. 5b. Under moderate noise (SNR ≈ 20), the RRR reconstruction exhibited pronounced amplitude distortions, whereas the gain-based solver produced a visually accurate approximation of **x**. This improvement is reflected quantitatively by the lower RSE values achieved by the gain-dissipative method, a performance gap that persists until around SNR ≈ 40. Above this threshold, the visual differences between the two solvers diminish, although residual discrepancies in ROE and RSE remain. Figure 5c summarizes the impact of noise on reconstruction accuracy, revealing that the gain-based approach and RRR perform similarly in the high-noise (SNR < 10) and low-noise (SNR > 40) regimes. Notably, however, the gain-dissipative solver holds a distinct advantage in the intermediate range 10 < SNR < 40, where the difference in RSE is both statistically and visually significant. In these conditions, the gain-dissipative system often recovers important structural details that RRR loses in noise.

**Fig. 6:**
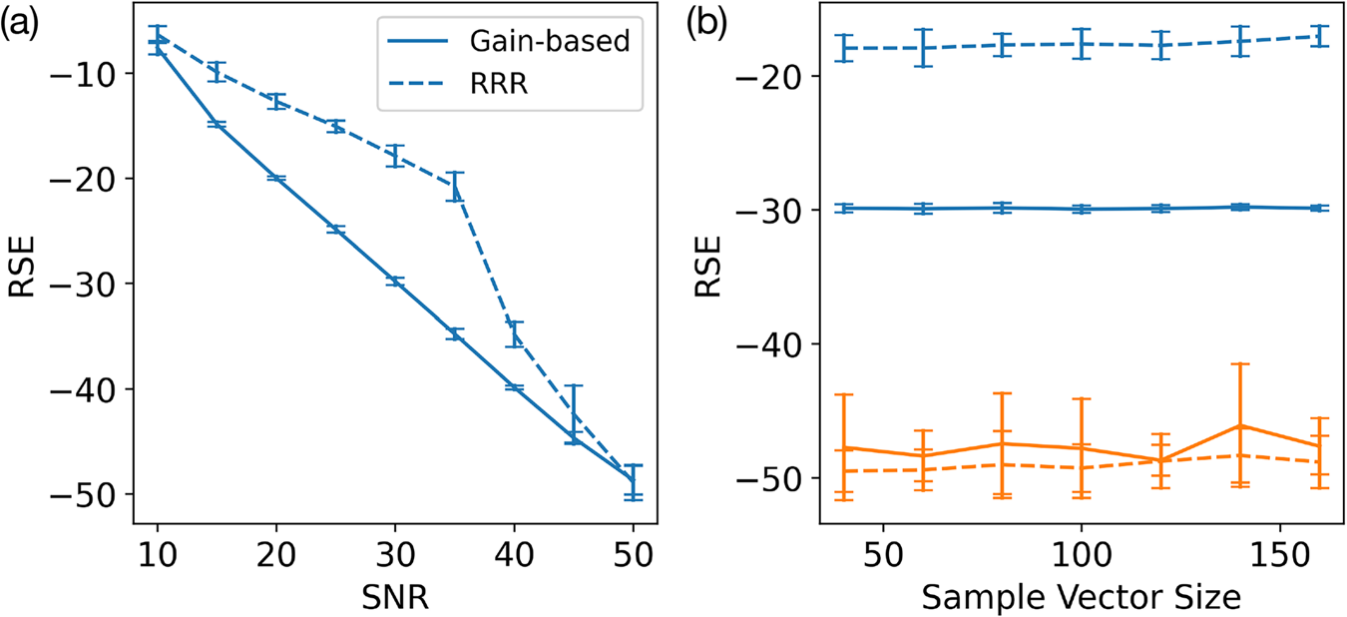
Phase-retrieval problems generated from random complex-valued samples with *L* = 5 phase filters. To solve each problem, the gain-based solver evolved to *t* = 1000, and RRR ran for 10,000 iterations. All noisy observation vectors $\widetilde{{{\bf{b}}}}$ contained Gaussian noise. **a** Phase-retrieval error (RSE) produced by the RRR method (dashed lines) and the gain-based system (solid lines) as a function of Gaussian noise in the measured amplitudes. At each noise level, 20 random complex samples were generated, each with 100 elements whose amplitudes are uniformly distributed in [0, 1) and phases in [0, 2*π*). The resulting observation vectors were then used for both methods. Vertical error bars indicate the standard deviation in RSE across the 20 trials. **b** Phase-retrieval error (RSE) versus the dimensionality of the sample vectors, comparing medium noise (SNR = 30, shown in blue) and low noise (SNR = 50, shown in orange). Solid lines again correspond to the gain-based system, and dashed lines correspond to RRR. Each data point represents the average RSE over 20 distinct random instances of the specified dimension, with error bars indicating the standard deviation. See “Fig. 6a” and “Fig. 6b” tables in supplementary data.

While the two-dimensional vortex example provides instructive insight into solver behavior at various noise levels, it remains a highly structured sample. To assess robustness on less structured data, we next consider *unstructured* samples **x**, whose amplitudes are drawn uniformly from [0, 1) and phases from [0, 2*π*). Keeping the dimensionality fixed and varying the noise level, we compared the RRR and gain-based methods, as summarized in Fig. 6a. The results largely mirror the vortex case: in the medium-noise regime, the gain-based solver attains markedly lower RSE than RRR, whereas both methods perform comparably under very high (SNR < 10) or very low (SNR > 40) noise. These findings indicate that the gain-based approach handles both structured and unstructured samples effectively, consistently outperforming RRR in the medium-noise band. Moreover, its RSE increases in tandem with SNR, suggesting stable performance across different noise levels.

All of the above tests involved samples of size 100. However, realistic applications typically require recovering much larger vectors. To explore how solution accuracy scales with dimensionality, Fig. 6b plots RSE against problem size for medium-noise (SNR = 30, in blue) and low-noise (SNR = 50, in orange) conditions, comparing the gain-based method (solid lines) and RRR (dashed lines). In both noise regimes, the RSE remains nearly constant even as the number of sample elements grows by a factor of four. This suggests that noise level influences the gain-based solver’s performance more than the underlying problem dimension. At very low RSE (~50 dB), the gain-based solver exhibits a slightly larger spread of RSE than RRR in Fig. 6. The spread can be explained by the following two effects. Firstly, the gain-based dynamics are integrated using an adaptive time-step ODE solver, so tiny variations in step acceptance or in the stopping time lead to small changes in the final state; when plotted in decibels, these tiny differences are amplified at very low decibel levels. Secondly, different initial conditions can relax into slightly different low-lying minima of the XY Hamiltonian. RRR iterations are discrete and extremely numerically stable, so the spread for RRR comes almost entirely from the choice of initial phases. Hence, the increased spread in RSE produced by the gain-based solver only appears deep in the regime of extremely small error, and does not indicate instability at moderate noise levels.

**Fig. 7:**
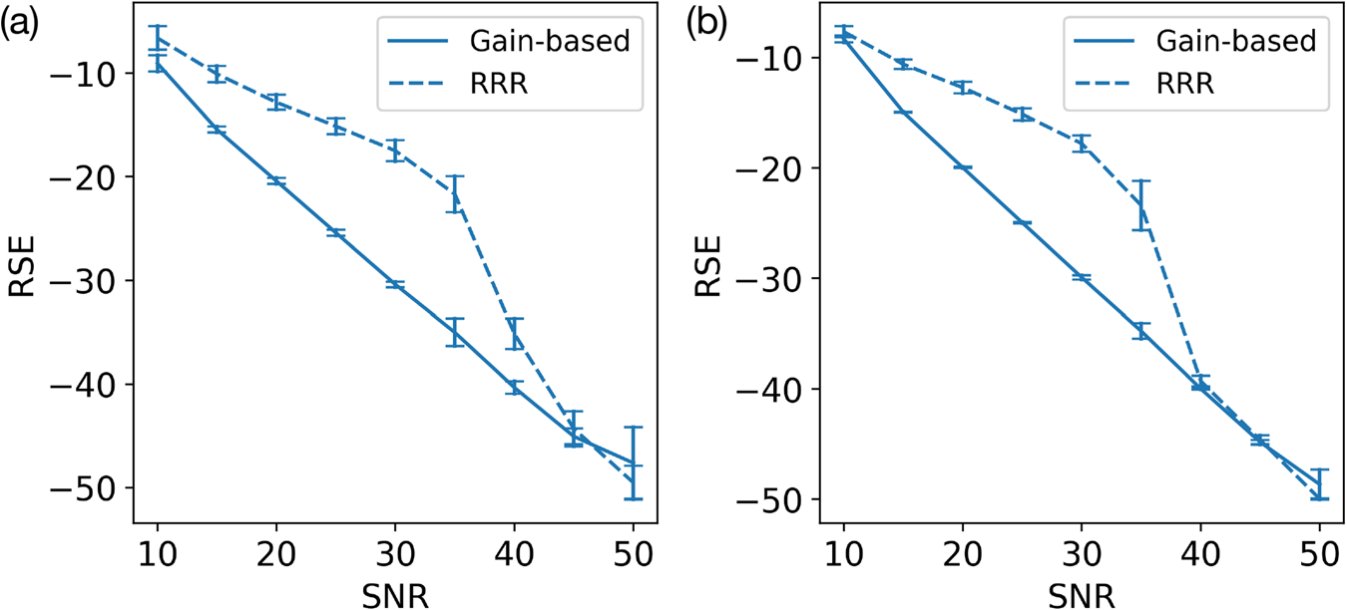
Phase-retrieval problems with Poisson or systematic errors. These problems were generated from random complex-valued samples with *L* = 5 phase filters and with Poisson noise or systematic errors added to observation vectors. For each problem, the gain-based solver evolved to *t* = 1000, and the RRR ran for 10,000 iterations. **a** Phase-retrieval error (RSE) produced by the RRR method (dashed line) and the gain-based system (solid line) as a function of Poisson noise in the measured amplitudes. **b** RSE produced by RRR and the gain-based methods as a function of systematic errors introduced into the measured amplitudes. In both panels, vertical error bars are the standard deviations in RSE across 20 different CDP phase-retrieval problems generated from random complex-valued sample vectors. These sample vectors had amplitudes uniformly randomly distributed in [0, 1) and phases uniformly randomly distributed in [0, 2*π*). See “Fig 7a” and “Fig. 7b” tables in supplementary data.

In addition, noisy observation vectors $\widetilde{{{\bf{b}}}}$ with Poisson noise or systematic errors, instead of Gaussian noise, were also presented to the solvers to reveal their robustness to different types of noise. Figure 7a shows the trend of solution quality measured by RSE as a function of Poisson noise magnitude measured by SNR, while Fig. 7b shows the RSE trend for observation vectors with constant systematic shifts to each of their elements. In both cases, phase-retrieval problems were generated from complex-valued sample vectors with random amplitudes in the range [0, 1) and phases in the range [0, 2*π*). Just like the Gaussian noise case shown in Fig. 6a, when the noisy observation vector $\widetilde{{{\bf{b}}}}$ contained Poisson noise or systematic errors, the gain-based method still clearly outperformed the RRR method under moderate noise, and performed similarly to the RRR method under low and high noise. This shows that the advantage of the gain-based method over the RRR method is robust to different kinds of noises that can be found in real diffraction experiments.

**Fig. 8:**
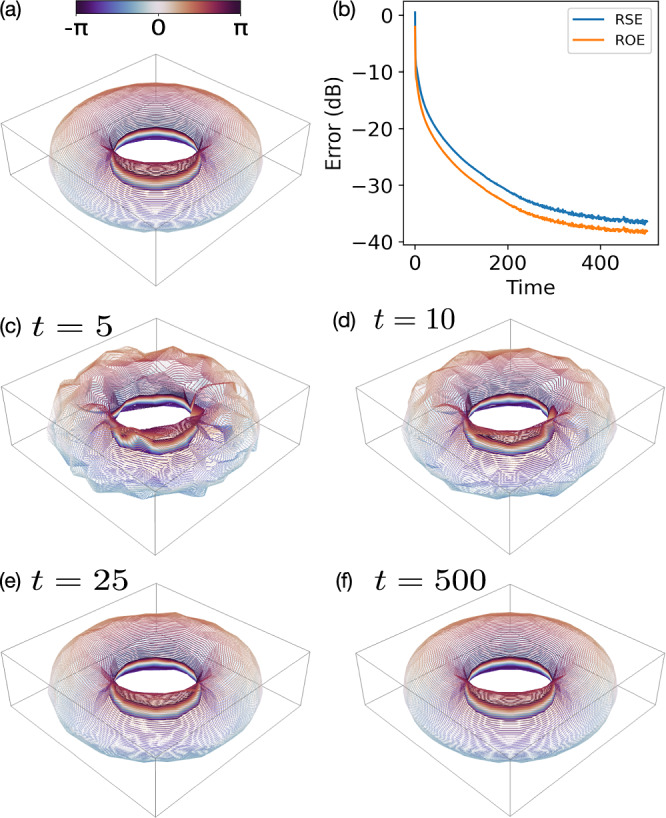
Three-dimensional vortex ring reconstructed using the gain-based phase-retrieval method. The phase-retrieval problem was constructed with *L* = 8 phase filters, and the field was discretised into a 21 × 21 × 7 grid, resulting in a sample vector **x** of size 3087, and an observation vector **b** of size 24,696. **a** Original vortex ring, visualized as an isosurface at 30% of its maximum amplitude. Phase isolines on this surface are colored according to their phase values, ranging from −*π* to *π*. **b** Time evolution of the phase-retrieval error (RSE and ROE) under gain-based dynamics. **c**–**f** Snapshots of the reconstructed vortex ring at *t* = 5, *t* = 10, *t* = 25, and *t* = 500, respectively, illustrating the progressive refinement of amplitude and phase in three dimensions. See “Fig. 8” table in supplementary data.

To illustrate how the gain-based method can recover phase information in cold-atomic Bose–Einstein condensate (BEC) experiments, where topological defects like solitons^47^, vortex lines^48^, and vortex rings^49,50^ naturally appear, we consider reconstructing a three-dimensional complex vortex ring. The sample vector is specified by

25 $$v(r,\theta ,z)\,=\,\frac{(r-{r}_{0})+i\,z}{\sqrt{{(r-{r}_{0})}^{2}\,+\,{z}^{2}\,+\,{\xi }^{2}}},$$

where (*r*, *θ*, *z*) are cylindrical coordinates, *r*_0_ is the ring’s radius, and *ξ* is the vortex-core size. Figure 8a shows an isosurface at 30% of the maximum amplitude, with phase isolines superimposed. The vortex flow, which is orthogonal to these lines, winds along the ring.

We construct a CDP phase-retrieval problem from this 3D complex-valued vector (3087 elements) and *L* = 8 phase filters, giving an observation vector **b** of size 24,696. We solved it using the gain-based system and Fig. 8b charts the time evolution of the retrieval error, while Fig. 8c–f depict snapshots of the reconstructed three-dimensional field at various stages. By the final state, the key vortex-ring features closely match the original, indicating that, with non-destructive CDP measurements, the gain-based approach can accurately recover the phase of 3D wavefunctions in BEC systems.

## Conclusion

We have shown that the coded diffraction pattern variant of phase-retrieval can be rigorously reformulated as an *XY* Hamiltonian minimization problem, paving the way for direct solution by gain-based oscillator networks and related physics-inspired systems. Through numerical tests, we demonstrated that such gain-based dynamics significantly outperforms the state-of-the-art Relaxed-Reflect-Reflect algorithm, particularly under medium-level noise (SNR values between 10 and 40 dB). Our findings hold for both structured data (e.g., two-dimensional vortices and three-dimensional vortex rings) and unstructured complex-valued data with random amplitudes and phases, and they are robust to Gaussian and Poisson noises as well as systematic errors in the observation vector.

Critically, we observed that the superior accuracy of the gain-based solver remains robust even as problem sizes grow. This scalability, combined with its noise resilience, indicates strong potential for large-scale real-world imaging tasks. Moreover, the gain-based approach can be physically realized in optical, polaritonic, or other nonlinear oscillator networks, thereby offering a hardware platform for rapid, energy-efficient phase retrieval. Such physical devices could perform continuous, parallel searches for global minima in the *XY* energy landscape, transforming this theoretical advantage into practical gains for real-time imaging and beyond.

Our gain-based algorithm requires only one continuous evolution of the oscillator network to a steady state for each CDP phase-retrieval problem, so the wall-clock time is essentially the physical relaxation time of the hardware. A summary of reported performance metrics of experimental implementations of various unconventional physical platforms could be found in ref. ^13^, and from there, we could provide some order-of-magnitude estimate of the expected computational speed of our gain-based algorithm when implemented in that hardware. In SPIMs, measured performance for ~10^2^ spins with 60 Hz liquid-crystal SLMs corresponds to a time-to-solution of order 2 s, dominated by the SLM refresh rate. Next-generation electro-optic SLMs with frame rates >1 GHz are projected to reduce this to ~10 μs for problems of similar logical size, since the optical evaluation of the XY energy is fully parallel in the number of spins. The SPIM platform has demonstrated all-to-all connectivity for spin counts up to ~10^5^ and projected capabilities near ~10^7^, making the CDP problem sizes studied here (*M* = *LN* ~ 2.5 × 10^4^–2.6 × 10^5^) well within the expected scaling regime. Coherent Ising machines (CIMs) report time-to-solution of a few milliseconds for problems of ~100 spins, suggesting that an *XY* implementation of Eqs. (12) and (13) would converge on sub-ms to ms timescales for the dimensionalities considered in this work.

Our results open a promising direction in the development of continuous-variable “*XY* machines”, enabling them to tackle large and noisy phase-retrieval instances that arise in a variety of scientific and industrial settings.

## Supplementary information

Description of the supplementary file

supplementary_data.xlsx

## Data Availability

The authors declare that all data supporting the findings of this study are available within the supplementary information files of this paper.

## Code availability

The code that supports the findings of this study is available from the first author (zw321@cam.ac.uk) upon request.
