# Supplementary material for: Phase retrieval via gain-based photonic XY-Hamiltonian optimization
Source: Commun Phys. 2026 Feb 3;9(1):85. doi: 10.1038/s42005-026-02525-7 (PMC12984011; doi:10.1038/s42005-026-02525-7)
Supplement: Supplementary file 1 — Description of the supplementary file [file 42005_2026_2525_MOESM1_ESM.pdf]

# Description of Additional Supplementary File

- File name: supplementary_data.xlsx
- `supplementary_data.xlsx` contains the data used to plot Figures 2–8.
